# Supplementary material for: An open-source framework for large-scale, flexible evaluation of biomedical text mining systems
Source: J Biomed Discov Collab. 2008 Jan 29;3:1. doi: 10.1186/1747-5333-3-1 (PMC2276192; doi:10.1186/1747-5333-3-1)
Supplement: Additional file 1 — Evaluation platform source code. The additional file contains the software evaluation framework discussed in this paper. The most current release can be downloaded from SourceForge.net [1]. See the accompanying README file for instructions on installation and use. Also included in the distribution is a collection of UIMA wrappers for some commonly used BioNLP tools and annotated corpora. [file 1747-5333-3-1-S1.zip › bionlp_uima_repository-1.0.1/docs/api/edu/uchsc/ccp/util/nlp/ei/xml/XMLEntityExtractor.html]

XMLEntityExtractor (Center for Computational Pharmacology Util/UIMA API)


|  |  |  |
| --- | --- | --- |
| **Overview** \| **Package** \| **Class** \| **Tree** \| **Deprecated** \| **Index** \| **Help** | |  |
| PREV CLASS   NEXT CLASS | **FRAMES**    **NO FRAMES**     **All Classes** |  |
| SUMMARY: NESTED \| FIELD \| CONSTR \| METHOD | DETAIL: FIELD \| CONSTR \| METHOD |  |


---


## edu.uchsc.ccp.util.nlp.ei.xml Class XMLEntityExtractor

```
java.lang.Object
  edu.uchsc.ccp.util.nlp.ei.xml.XMLEntityExtractor
```

---

``` public class XMLEntityExtractor extends Object ```

---

**Constructor Summary**

`XMLEntityExtractor()`


| **Method Summary** | |
| --- | --- |
| `static List<TextAnnotation>` | `parseEntitiesFromSparseUntokenizedXML(String taggedText, String openTagText, String closeTagText, String documentID, int documentCollectionID, String classMentionType, Annotator annotator, AnnotationSet annotationSet)`             Use when trying to get entity spans from xml that has not been tokenized (you therefore do not need the original text) NOTE: Due to the primitive nature of this code, be sure to remove all xml tags except for the one you are looking for. |
| `static List<TextAnnotation>` | `parseEntitiesFromSparseUntokenizedXML(String taggedText, String openTagText, String closeTagText, String documentID, int documentCollectionID, String classMentionType, Annotator annotator, AnnotationSet annotationSet, boolean removeInternalXMLTags)` |
| `static List<TextAnnotation>` | `parseEntitiesFromSparseXML(String taggedText, String originalText, String tag, String documentID, String classMentionType, Annotator annotator)` |

**Methods inherited from class java.lang.Object**

`clone, equals, finalize, getClass, hashCode, notify, notifyAll, toString, wait, wait, wait`

**Constructor Detail**

### XMLEntityExtractor

```
public XMLEntityExtractor()
```


**Method Detail**

### parseEntitiesFromSparseXML

```
public static List<TextAnnotation> parseEntitiesFromSparseXML(String taggedText,
                                                              String originalText,
                                                              String tag,
                                                              String documentID,
                                                              String classMentionType,
                                                              Annotator annotator)
```

---


### parseEntitiesFromSparseUntokenizedXML

```
public static List<TextAnnotation> parseEntitiesFromSparseUntokenizedXML(String taggedText,
                                                                         String openTagText,
                                                                         String closeTagText,
                                                                         String documentID,
                                                                         int documentCollectionID,
                                                                         String classMentionType,
                                                                         Annotator annotator,
                                                                         AnnotationSet annotationSet)
```

:   Use when trying to get entity spans from xml that has not been tokenized (you therefore do not need the original text)
    NOTE: Due to the primitive nature of this code, be sure to remove all xml tags except for the one you are looking for.

    :   **Parameters:**: `taggedText` -: `openTagText` -: `closeTagText` -: `documentID` -: `documentCollectionID` -: `classMentionType` -: `annotator` -: `annotationSet` - **Returns:**

---


### parseEntitiesFromSparseUntokenizedXML

```
public static List<TextAnnotation> parseEntitiesFromSparseUntokenizedXML(String taggedText,
                                                                         String openTagText,
                                                                         String closeTagText,
                                                                         String documentID,
                                                                         int documentCollectionID,
                                                                         String classMentionType,
                                                                         Annotator annotator,
                                                                         AnnotationSet annotationSet,
                                                                         boolean removeInternalXMLTags)
```


---


|  |  |  |
| --- | --- | --- |
| **Overview** \| **Package** \| **Class** \| **Tree** \| **Deprecated** \| **Index** \| **Help** | |  |
| PREV CLASS   NEXT CLASS | **FRAMES**    **NO FRAMES**     **All Classes** |  |
| SUMMARY: NESTED \| FIELD \| CONSTR \| METHOD | DETAIL: FIELD \| CONSTR \| METHOD |  |


---
